# Artificial neural network based predictive modeling of viscosity of an oil based hybrid nanofluid

**DOI:** 10.1016/j.mex.2025.103748

**Published:** 2025-12-04

**Authors:** Muhammad Furqan, Fahim Raees, Muhammad Khalid

**Affiliations:** a Department of Mathematics, NED University of Engineering and Technology 75270 Karachi, Pakistan; b Department of Physics University of Karachi 75270 Karachi, Pakistan

**Keywords:** Viscosity, carbon nanotube, Engine oil 20w-50, Nanofluid, Artificial neural network

## Abstract

In many industries like micro-electronics, mechanical engines, transportation, manufacturing and nuclear reactors cooling characteristics which is a crucial challenge can optimize using nanofluid. Nanofluids are also used for better energy transfer and storage in many devices. In the current study the viscosity variation studied by the impact of input variables solid volume fraction and temperature. Sol-gel auto-combustion was used to prepare nickel ferrite (NiFe_2_O_4_) nano-particles. The structure of the NiFe_2_O_4_ was obtained using an X-ray diffraction method. The Fourier transform infrared spectrum provided evidence of NiFe_2_O_4_ presence. In order to better understand the surface morphology of nickel ferrite nano-particles, scanning electron microscopy (SEM) was employed. AT the various concentrations of nanofluid 0, 0.25, 0.50, 0.75, and 1 % were prepared. The viscosity was calculated at temperatures ranges 40 to 80 °C. The experimental results showed that the viscosity of nanofluid (µ_nf_) falls as the volume fraction(φ) increases and as the temperature rises. RSM and ANN predicted data have R^2^ values of 0.9987 and 0.9999 respectively.. ANN accuracy can also be seen by MSE value. ANN shows less MSE value (0.00001) as compare to RSM that has MSE 0.0002. Which shows the ANN accuracy over RSM.

Predictive modelling for viscosity of Oil-Based Hybrid Nanofluid.

Artificial Neural Network and Response surface methodology methods were used for correlation.

Results were correlated with experimental data.

## Graphical abstract

**Figure:**
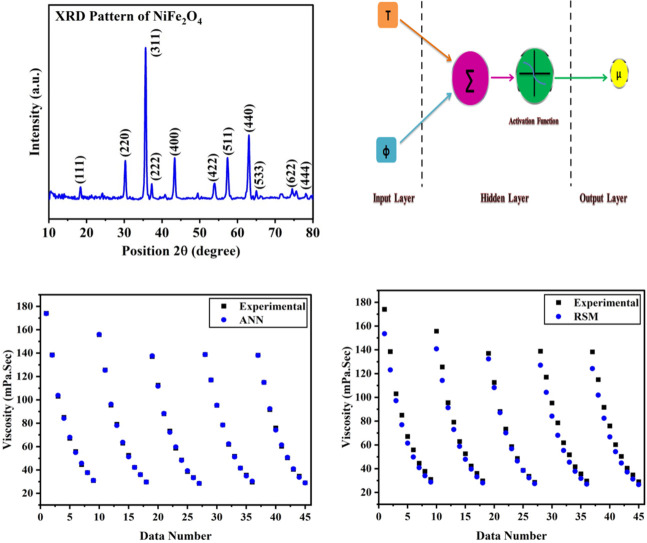

## Specifications table

**Table:** 

**Subject area**	Mathematics and Statistics
**More specific subject area**	*Fuzzy logic*
**Name of your method**	*Artificial Neural Network and Response Surface Methodology*
**Name and reference of original method**	*Not Applicable*
**Resource availability**	*Not Applicable*

## Background

Heat transfer plays a crucial role in industrial and power plant operations, driving continuous research to enhance heating equipment efficiency [1]. Nanofluids, which are base fluids containing nano-sized particles, have gained attention for their exceptional thermal properties. Despite numerous studies on nanofluid’s thermal conductivity, there is limited research focused on their viscosity, particularly under high-temperature conditions. Most studies use base fluids like water or ethylene glycol, which are unsuitable for high-temperature applications ([2,3]). Understanding the viscosity of nanofluids it is important for thermal energy storage materials in energy-harvesting technologies such as concentrating solar power (CSP) plants, where molten salt eutectics improve overall performance.

Engine oil is another key lubricant used in many industries, especially automotive engines, where temperature fluctuations significantly affect oil viscosity. In hot climates, engine oil thins, reducing its ability to form protective films and increasing engine wear [4]. Oils like 20W-50, known for their higher viscosity, are preferred for hot conditions and heavy-duty engines. Enhancing engine oil’s thermal conductivity while managing viscosity is vital to improving engine cooling and reducing friction.

Nanofluids incorporating materials such as carbon nanotubes (CNTs) and metallic nanoparticles have shown promise in increasing thermal conductivity. For instance, the nanofluid NiFe2O4-MWCNT/20W-50 demonstrates optimal thermal characteristics for vehicles operating in hot environments. Studies reveal that adding nanoparticles to molten salts greatly boosts their thermal conductivity, making nanofluids an economical option for electricity generation. The present research focuses on the viscosity behavior of high-temperature carbon nanotube-based nanofluids, specifically hybrid nano-lubricants like MWCNTs/NiFe_2_O_4_ combined with engine oils 20w-50.

The viscosity of nanofluid depends on several factors, including temperature, nanoparticle concentration, and size. Classical models such as those proposed by Batchelor and Wang ([5,6]) describe viscosity as a function of particle volume fraction but do not fully account for temperature variations or nano-scale effects. Hybrid nanofluids, which combine two types of nanoparticles, have garnered interest due to their enhanced thermo-physical properties. Examples include mixtures such as Cu/Al_2_O_3_-water, MWCNT/SiO2-water, and MWCNT/Fe2O3-water, among others, showing improvements in both thermal conductivity and viscosity.

Recent studies have examined the effects of nanocomposites on heat transfer.

[7] et al. used the Galerkin finite element method to study heat transport in a tetra-nanofluid between a converging and a diverging channel under the impact of thermal radiations. This paper reports on the study of a tetra nanofluid and similarity transforms within a converging/diverging channel under the novel effects of thermal radiations and dissipation functions. The Galerkin Finite Element Method (GFEM) is used to provide the results. Due to their superior thermal conductivity, tetra nanofluids are found to have a higher temperature capability than ternary nanofluids. This conclusion is supported by comparative impacts of thermophysical models under various volume concentrations, which also show that tetra nanofluids are superior to conventional nanofluids.

[8] et al. used thermal slip and dissipation function to investigate the significance of radiated ternary nanofluid for thermal transport in stagnation point flow. The model used in this work predicts the parametric ranges for better heat transfer results and includes the motivating elements of thermal slip, convective condition, radiative flux, and viscous dissipation. The amount of Al_2_O_3_, CuO, and Cu nanoparticles is maintained at 0.04. The shooting scheme-based numerical technique is used to solve the model and simulate the outcomes. This study discovered that when a ternary nanofluid has a thermal slip number of α_3_=0.1,0.2,0.3,0.4, the temperature is greater. The temperature was effectively raised by the Biot number in the range of B_1_=0.1,0.3,0.5,0.7, and it disappeared after η=1.5, which is a physical representation of the ambient location from the surface. The temperature near the surface increased significantly with the addition of Ecert number effects for rising values from Ec=0.1,0.2,0.3,0.4, and is optimal for the case of ternary nanofluids. Al_2_O_3_ concentration (φ_1_=0.01,0.02,0.03,0.04) and the impacts of thermal radiation (Rd=0.1,0.3,0.5,0.7) generated heat in the fluid, which is why the total temperature in nano, hybrid, and ternary nano liquids was at its highest. While ranging from 0.1, 0.3, 0.5, and 0.7, the heat transfer rate (Nusselt number) is shown to be optimal for thermal slip, Biot, and radiation number. A high concentration of cumulative nanoparticles up to 0.04 has a direct effect on the functional fluids' thermal conductivity, which may be important for thermal enhancement applications.

[9] et al. worked on the manuscript that covered the subjects of magneto-nanofluid flow between two parallel disks that is time dependent. Additionally, the consequences of viscous dissipation were considered. In the base fluid, three distinct shaped nanoparticles were suspended. For the analysis, Hamilton and Crosser's model for the nanofluid's effective thermal conductivity had been taken into account. The Brinkman model for dynamic viscosity was applied. The resulting problem was solved using the Runge-Kutta numerical approach, Variation of Parameters approach (VPM), and Adomian's decomposition method (ADM). This study examined the effects of the nanoparticle volume percentage and Hartmann number on velocities (both in the x and y directions). It has been discovered that the temperature field rapidly drops for platelet-shaped nanoparticles because of their variable Hartmann numbers, and that the temperature drops for nanoparticles with a volume fraction that is nearly insignificant. For platelets, cylinders, and brick-shaped nanoparticles, the skin friction coefficient and local rate of heat transfer are computed numerically.

[10] et al. worked on the Analytical Study of the Blood/Graphene Hybrid Nanofluid Influenced by Joule Heating via VIM and Platelets-Cylindrical Nanoparticles. A novel biohybrid nanofluid model (BHNFM) including (Ag− *G*) hybrid nanoparticles and the important physical effects of resistive heating, magnetic field, and quadratic radiation were used in this investigation. In this modal, the variational iteration method was used. This study found that the biohybrid nanofluid (BHNF) is preferable to mono-nano BHNFs in terms of regulating fluid flow. This study found that the increased viscous forces brought on by the composite density of Ag−*G* cause the velocity of BHNF to be lower than that of MBNF (mono bionanofluid). By applying a strong magnetic field to the channel, the mobility of BHNF and MBNF is significantly decreased. By optimizing the heating species and quadratic radiations, the temperature of BHNF and MBNF rises.

[11] et al. worked on a magnet-based geometric thermal study of radiated (aluminum oxide)/water using the CattaneoChristov and Corcione models. In this work, a novel model was formulated using improved nanofluid characteristics and physical parameters. The results were then thoroughly investigated mathematically using a numerical approach, and the results were examined for important physical parameters. By increasing the particle concentration to φ=0.6 %, Corcione's model boosted the estimation of thermal conductivity, while electrical conductivity decreased from 0.999001 to 0.994036. Additionally, the results showed that the nanofluid movement is slow compared to conventional liquid due to high viscous forces, and the stretching number α_1_ from 0.1 to 1.3 and α_2_=0.1,0.2,0.3,0.4 resist fluid flow across the surface. Additionally, the model is more applicable for high thermal transport applications when radiative thermal flux (Rd) and internal heating effects (Hg) are included. This is especially true for nanofluids. Maintaining Rd and Hg between 0.0 and 0.8 could increase the local thermal rate at the surface. For simple fluids, the rate of heat transmission is small, but for nanofluids, it is dominant.

[12] et al. Studied the dynamics of Corcione nanoliquid on a convectively radiated surface using Al_2_O_3_ nanoparticles. In this work the model aims and designed to investigate the heating characteristics of Al_2_O_3_/H_2_O and the effective values of estimated using Corcione model. The model equations included the effects of internal heating sources and thermal radiations. The model results were calculated and thoroughly examined using the RK (Runge–Kutta) technique. Additionally, it was discovered that the increased Hartmann number had a significant impact on the motion of the particles and that, because of the stronger viscous forces, the nanofluid moved quickly. Furthermore, by taking into account the Corcione model, the heating source and thermal radiations raised the temperature of the nanoliquid.

[13] et al. investigated an unstable nanofluid over a half-infinite domain using the Effective Prandtl Number Model (EPNM) and parametric influences. The performance of Al_2_O_3_/H_2_O may be influenced by the up to quadratic nanoparticle concentration factor included in the EPNM model. Because of their potential qualities, Al_2_O_3_ nanoparticles have become very popular. As a result, this study used a revolving disc to do a fresh, innovative model-based analysis. Brakes, gears, gas turbines, flywheels, and other devices use this geometry extensively. The fundamental physical components, such as an exponentially rising heat source, a normal magnetic field, Joule heating, etc., are included to increase the model's dependability for a variety of applications. After that, a thorough numerical analysis of the model is carried out, and the findings are interpreted. Analysis was done on the effects of Al_2_O_3_ concentration on the properties of the nanofluid, thermal behavior, shear drag, and heat transport rate.

[14] et al. investigated aggregation and nanolayer effects regarding the nanoparticles. Aspects of Joule heating and irregular heat source in Fe_3_O_4_/C_2_H_6_O_2_ nanofluids for magneto-radiative heat transfer. Magnetite nanoparticles (NPs) give Fe_3_O_4_/C_2_H_6_O_2_ better thermal conductivity, which raises cooling systems' thermal efficiency. Using a thin needle setup, this work intends to investigate the transport characteristics of Fe_3_O_4_/C_2_H_6_O_2_ in addition to nonlinear radiations, magnetic fields, dissipation, Newtonian heating, and nonuniform sources. Multiple findings are produced numerically across the domain using two different types of models based on aggregated NPs and NLNPs (nanolayer nanoparticles) of nonlinear character. Through graphical findings, the models' relative performance is provided, and nonlinear radiations and dissipation are studied.

[15] et al. investigated radiating nanofluids (SiO_2_/H_2_O, Al_2_O_3_/H_2_O) under mixed convection using AI neural networking (LMBPS). A thorough comparison of, Al_2_O_3_/H_2_O and SiO_2_/H_2_O employing exponential and quadratic type features was carried out in this work. The results were obtained using an AI-based approach, and the data was obtained using the bvp4c method. After that, the LMBPS was used for regression, functions fit, histogram error, state, and training performance. For φ = 0.01 t0 0.04, it was shown that Al_2_O_3_/H_2_O is more effective than SiO_2_/H_2_O, which increased the fluid's functionality and thermal conductivity. When the Biot number rises and the effects of changing α are less pronounced, a very high performance is evaluated.

[16] et al. investigated the importance of Fe_3_O_4_/MnZnFe_2_O_4_ nanoparticles for ethylene glycol's thermal efficiency. CattaneoChristov theory analysis for single-phase nanofluid flow. In this work, ethylene glycol served as the main solvent for heat transfer applications using the hybrid nanofluid Fe_3_O_4_/MnZnFe_2_O_4_. In addition to nanoparticles, physical phenomena such as heating species, CattaneoChristov thermal flux, and surface temperature variation may also affect how well single phase nanofluids work. Thus, the current endeavor seeks to define a single-phase problem using affected parametric ranges and a slanted elongating surface with an acute angle with ground level. After a numerical analysis of the last problem (shooting scheme combined with RK scheme), the ranges for improved performance were projected and studied.

Vakili-Nezhaad et al [17] analyzed the influence of MWCNTs on viscosity indices of lubricating oil, while Vasheghani et al [18] studied Al2O3’s role in enhancing motor oil’s properties. Others have investigated hybrid nanoparticles like SiO2/MWCNT and their effect on engine oil viscosity. Despite these advances, no prior research has explored the viscosity of MWCNTs/ZnO-SAE40 hybrid nano-lubricants [19,20].

Cooling the devices is one of the one of the most engineering challenges. Optimization of heat transfer in fluid flow is crucial in applied industries. Increasing the efficiency of energy conversion systems requires improving their thermal performance. Through the nanofluid we can overcome the cooling challenges. Hybrid Nanofluid (NiFe_2_O_4_−MWCNT) was used in our study. NiFe_2_O_4_ have many useful characteristics (magnetic, catalytic, energy storage and biomedical) While MWCNT (Multiwall Carbon Nano Tubes) have high elastic properties, energy storage, high mechanical strength and stiffness, excellent electrical and thermal conductivity, good chemical and thermal stability and a high aspect ratio. So, the hybrid nano particles for engine oil 20 W-50 have more colloidal suspension than other fluids. This paper aims to fill that gap by experimentally measuring the absolute viscosity of MWCNTs/NiFe_2_O_4_–20w-50 hybrid nano-lubricants across various concentrations and temperatures. Additionally, it proposes a predictive correlation for the dynamic viscosity of this hybrid nanofluid, employing artificial neural networks and response surface methodology. The goal is to better understand viscosity changes with temperature and particle concentration, aiding the design of efficient lubricants and coolants for vehicles operating in hot climates. One of the novelties of the study is the preparation of efficient nanofluids with engine oil as base fluid with optimum viscosity, which have the most beneficial application in many industrial sectors of many types of engines, on the other hand predictive modeling of viscosity of hybrid nanofluid that made easy to simulate the optimum results with the real-world applications of oil industry to overcome the cost of characterization and errors in making samples and characterization.

## Method details

### Experimental procedure

#### Fabrication of nano-particle

To begin this stage, multiwall carbon nanotubes and nickel spinel ferrite nano-particles were used separately. Then, combine 80 ml ortho-xylene and 20 ml oleic acid in a volume-to-volume solution. Stirred a result for many hours to make it homogeneous. On the other hand, for surface modification, 2 g of nickel ferrite nano-particles were added to 98 g of ortho-xylene, and the mixture was continuously stirred. At the same time, one places the container on a hot plate. The surface-modified nano-particles are then filtered off, and the particles are added to the oleic acid solution. A dry surface-modified nickel nano-particle powder remained after heating the liquid with a hot plate.

#### Characterization

An XRD investigation supported multi-carbon nanotubes (MWCNT) and nickel ferrite (NiFe_2_O_4_) crystal structures. Energy dispersive x-ray spectroscopy (EDX) verified the composition of (NiFe_2_O_4_) and (MWCNT). The X-ray diffraction method and an X-ray diffractometer were used to examine the crystalline structure of the prepared nano-particle. To get the XRD spectra, Cu-Kα radiations having a wavelength of 1.54 Å were passed through powdered nano ferrites. One obtained the XRD studies at room temperature with '2θ' ranging between 10 and 80 degrees. The NiFe_2_O_4_ phase is represented by the spectrum peaks at (111), (220), (311), (222), (400), (422), (511), (440), (533), (622), and (444), which imply a cubic structure of spinel. Scherer's formula is used to compute the average crystallite size of nickel ferrite.

(1) $$D=\frac{0.693\lambda }{\beta cos\theta }$$

**Fig. 1:**
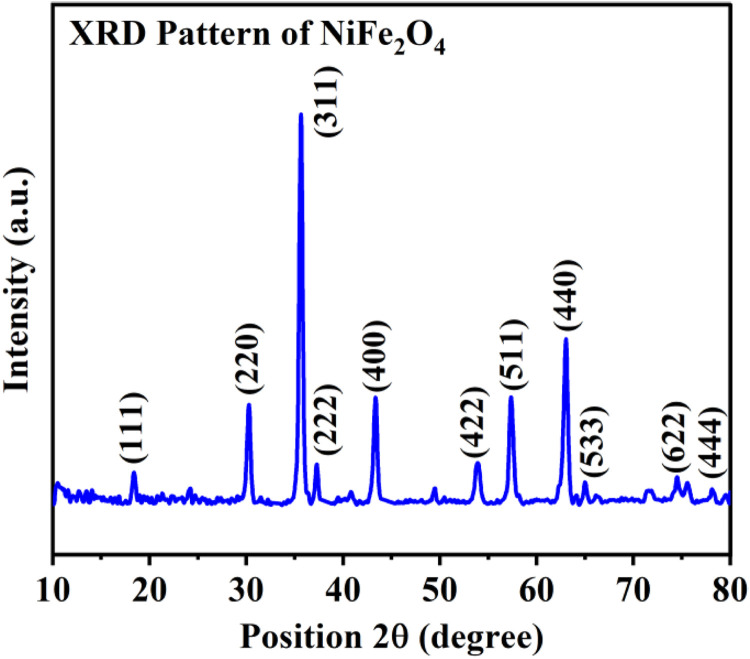
The synthesized nickel ferrite nano-particles X-ray diffraction pattern.

The XRD pattern of NiFe_2_O_4_ showed in Fig. 1. The X-ray wavelength, which is 0.15405 nm, is represented by λ, the angle at the highest peak by θ, the full breadth and half maximum by β, and the average crystallite size in nanometers by D. The maximum intensity in the X-ray diffraction analysis is observed at 35.8 degrees. According to the Scherer Eq. (3), the mean crystallite size is approximately twenty nm.

**Fig. 2:**
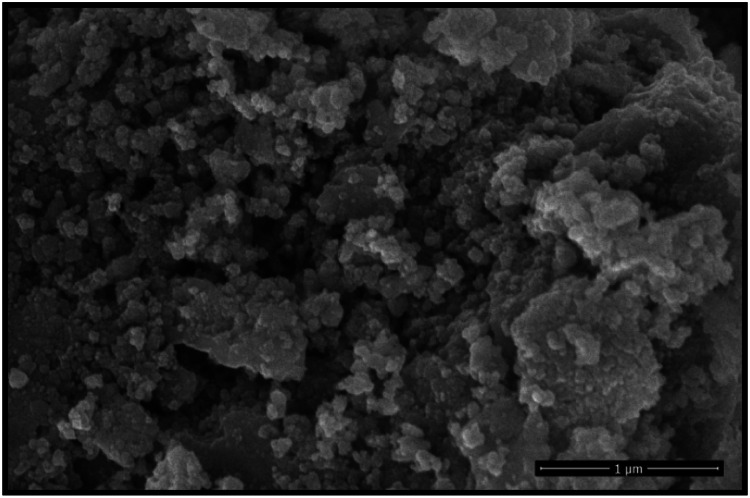
The above picture shows the Scanning electron microscopic image of Nickel Ferrite nano-particles.

One of the most common approaches for analyzing the surface morphology of nano-particles is scanning electron microscopy (SEM). The signals generated by electron-sample interactions reveal the material's surface topography (pattern) and chemical properties. Fig. 2 displays the surface structure of the produced nano-particles.

#### Preparation of nife_2_o_4_−MWCNT nanofluid

**Fig. 3:**
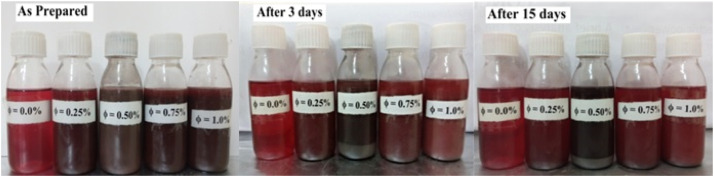
The above image represents a hybrid nanofluids sample.

Nickel ferrite oxide dry powder was mixed with MWCNT and then 20W-50 SAE engine oil in a specific ratio to make different samples with varying solid volume fractions (SVF) such as 0, 0.25, 0.50, 0.75, and 1.0 %. Using the relationship mentioned in Eq. (4) and the weight of the NiFe_2_O_4_−MWCNT and engine oil, which are both specified in (Table 1). Using several compositions of hybrid nanofluid, we made five sample series, as shown in Fig. 3, stirring the samples constantly to check for nano-particle stability in a nanofluid. The particle stability in samples is mentioned in (Fig. 3).

(2) $$\varphi \left(\%\right)=\;\frac{m_{NF}}{m_{NF}+m_{OIL}}\times \;100$$

**Table 1:** Composition of nanofluid.

Sample No	Solid Volume Fraction ( %)	Nano-particle mass m_NF_ (g)	Base Fluid mass m_OIL_(g)
1	0.00	0.000	49.830
2	0.25	0.1375	49.690
3	0.50	0.275	49.550
4	0.75	0.4125	49.005
5	1.00	0.550	49.770

For each sample, 60 ml of nanofluid was prepared using hybrid nano-particles and oil.

The basic oil was manually blended with hybrid nano-particles. The base oil and nano-particle mixture was placed in an ultrasonic bath for eight hours to homogenize and break up the particle lumps. For this, the UC-4120 model of an ultrasonic bath was employed.

### Viscosity measurements

Viscosity was measured using the falling ball method throughout a temperature range of approximately 40 to 80 degrees Celsius. Even though temperature directly affects nanofluids’ viscosity, we used a circulating temperature control system to maintain sample temperatures throughout the experiment with an accuracy of 0.1 °C. It was carried out using the LAUDA Alpha LCE 0226 temperature bath. Experimental methods are used to determine the prepared samples' viscosity.

(3) $$\eta =\mathrm{KT}\left(\rho_{1}-\rho_{2}\right)$$

Where ρ_1_ and ρ_2_ are the densities of the spherical ball and the hybrid nanofluid, respectively, and K is the proportionality constant, T is the time for which the free-falling spherical ball spends passing through the nanofluid between two fixed positions in Eq. (5). Fig. 4(a), Fig. 4(b) show the relationship between the experimentally estimated viscosity of nanofluids and the input parameters temperature and solid volume fraction.

**Fig. 4(a):**
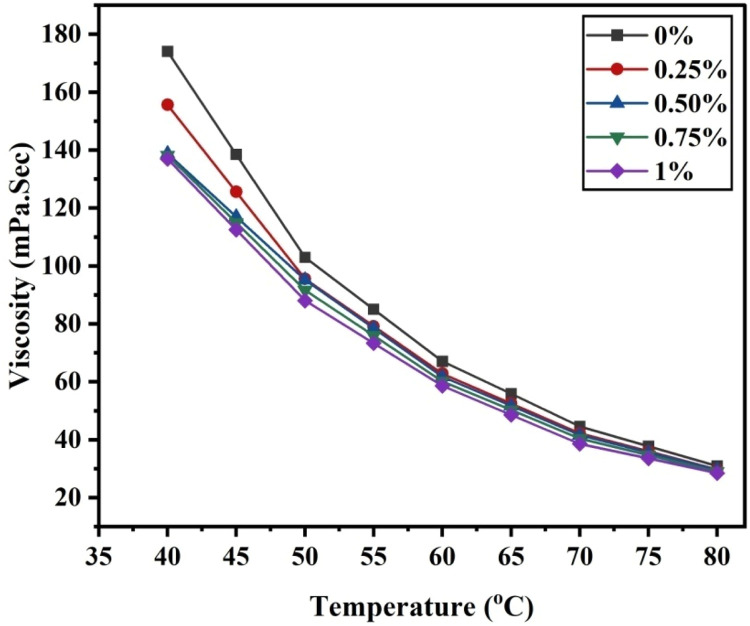
The effect of temperature on nano lubricant viscosity at various SVF.

**Fig. 4(b):**
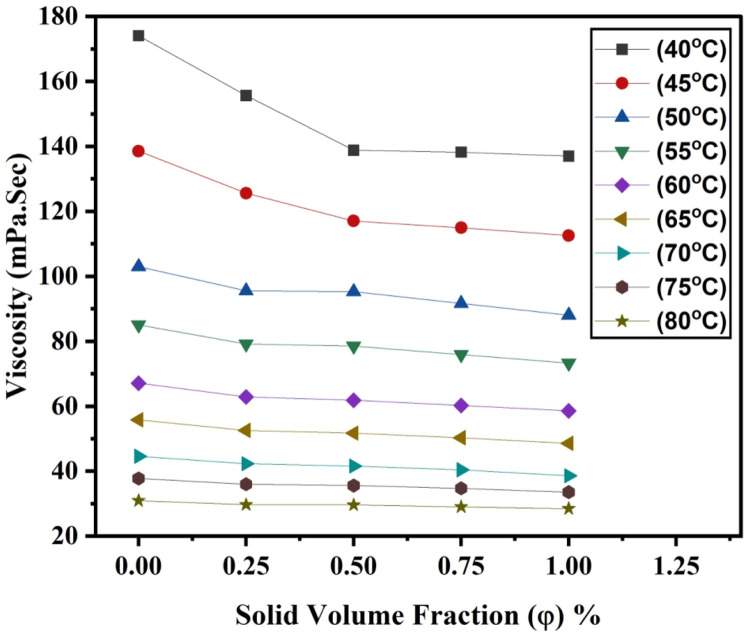
The effect of SVF on nano lubricant viscosity at various temperatures.

## Theoretical models

### Artificial neural network (ANN)

#### ANN architecture

One kind of machine learning method used for adaptive control, predictive modeling, and problem solving in artificial intelligence is the artificial neural network. ANNs may learn from data because they are composed of linked layers of nodes, or neurons, that process information in a way similar to that of the human brain.

The architecture of ANN consists of three common layers.

i. Input Layer
ii. Hidden Layer
iii. Output Layer

#### Input layer

It is the first layer of the network and it receives the data, which usually is in the form of vectors. The vector may contain any number of parameters. Generally, the number of input nodes in the input layer is equal to the number of parameters in the input vector. Input layers preprocess the data and feed it to subsequent hidden layers. The input nodes do not change the data; it simply checks if the data is in a valid format and then passes it along to the next layer.

#### Hidden layer

The hidden layers are where most of the processing takes place. The number of hidden layers may differ. The hidden layer multiplies the input values with weights as the incoming data moves through weighted connections. The weighted inputs are then added together to generate a single number.

#### Output layer

The output layer receives the information that has been processed. Either the input layer, the hidden layer, or both may be connected to the output layer. In certain instances, the input layer receives information from the output layers. The final prediction value is produced by the output layer. Classification networks usually include a single output node. To generate the output values, the activation functions at the output layer's nodes add and modify the data. For neural networks to identify meaningful data patterns and avoid overfitting, proper weight modification is essential.

#### ANN configuration

**Fig. 5:**
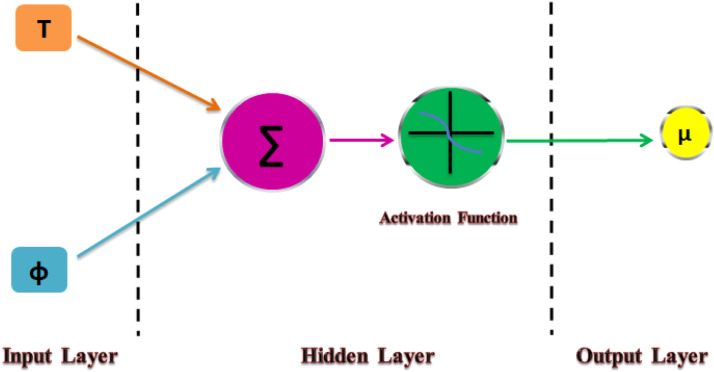
Schematic diagram of detailed explanation of entire ANN process.

The accuracy of this method in function approximation has been shown in several investigations. Various tactics are used, and overall performance is compared due to the efficacy and efficiency of backpropagation techniques during this artificial neural network training. Schematic diagram of detailed explanation of entire ANN process has been described in Fig. 5. Numerous artificial neural networks were examined in the buried layer while considering different neuron counts and transfer functions. The ideal topology was discovered through trial and error by decreasing the ANN error. Utilizing two neurons in the second layer with hyperbolic functions in the output layer and linear functions in the input layer led to a novel result. Table 2 describes the entire ANN topology configuration. A SAE 20W-50 hybrid nanofluid was divided into 45 samples based on temperature and volume concentrations.

**Table 2:** The ANN configuration.

Networking Parameters	Explanation
ANN design	Perceptron with many layers
Training approach	Backward Propagation
Error parameter	Average square deviation
Optimum training method	trainbr
No. of Hidden layer functions	2
Hidden layer functions	tan-sig and log-sig
Output layer functions	Pure-lin
No. of training result	31
No. of the validation result	7
No. of test result	7

The raw data was typically divided into three portions: 70 % for training, 15 % for validation, and 15 % for testing. The artificial neural network with the most significant result is excellent for system simulations (lowest error level). For each ANN, various topologies (number of hidden layer neurons and transfer functions) have been investigated, and the most effective combinations have been determined through trial and error. This is because the population of neurons in the hidden layer significantly impacts modelling accuracy. The distinctive results are obtained using two neurons in the second layer and a linear transfer output layer with a tangent-hyperbolic sigmoid function. Methods for instruction One of the novel features of this study was identifying the most effective training method for an artificial neural network that is significantly more stable while still being able to estimate the optimum viscosity performance. For several reasons, Strategies for forward-based training disclosed the desired value and the actual output's mean square error (MSE) as shown in Eq. (6). The output of a top-notch artificial neural network should be precise and contain the intended value. The theoretical correlation and slope values must be equal, and the biased value must be zero. The regression line's slope is nearly equal to 1 in each of the four plots, suggesting that the ANN output values were calculated accurately enough to provide the intended outcome. Additionally, all of the points are on the plane's bisector, and the dispersion style of the all-data point appears to be at its lowest level. Finally, a modified artificial neural network examines the viscosity of SAE 20W-50 engine oil nano-fluids at various temperatures and nano-particle composition.

An artificial neural network's optimal architecture is found through trial and error. The tan-sig function and two layers with two neurons inside the hidden layers produced the best ANN performance in this study. Furthermore, the trainer approach generates a well-trained ANN for μ_nf_ prediction with a correlation coefficient 0.9999 and an MSE of 0.00001 or 1 × 10^–5^. The equation below illustrates the mean square error, where N is the quantity of the number of data points, y_exp_ is the actual value, and Y_ann_ is the targeted value, as an artificial neural network.

(4) $$MSE=\frac{1}{N}\sum_{i=1}^{N}{\left(y_{\exp-y_{ANN}}\right)}^{2}$$

**Fig. 6:**
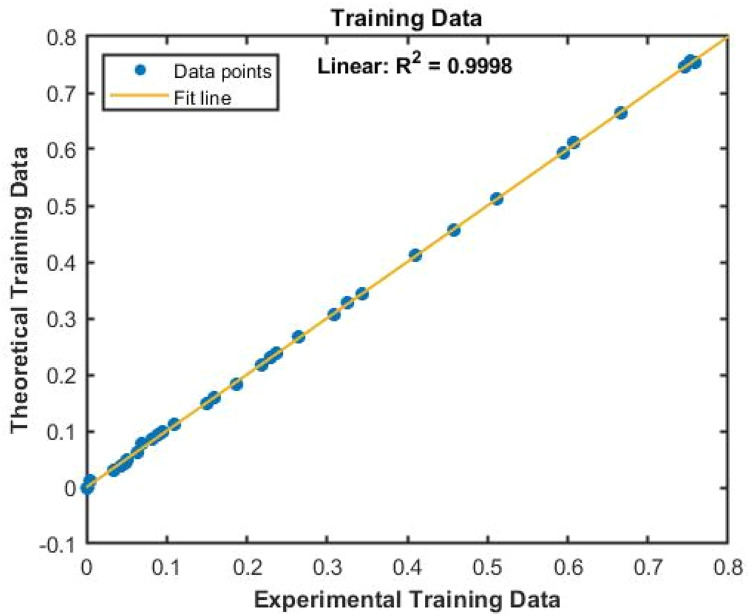
Experimental training data against theoretical training data.

**Fig. 7:**
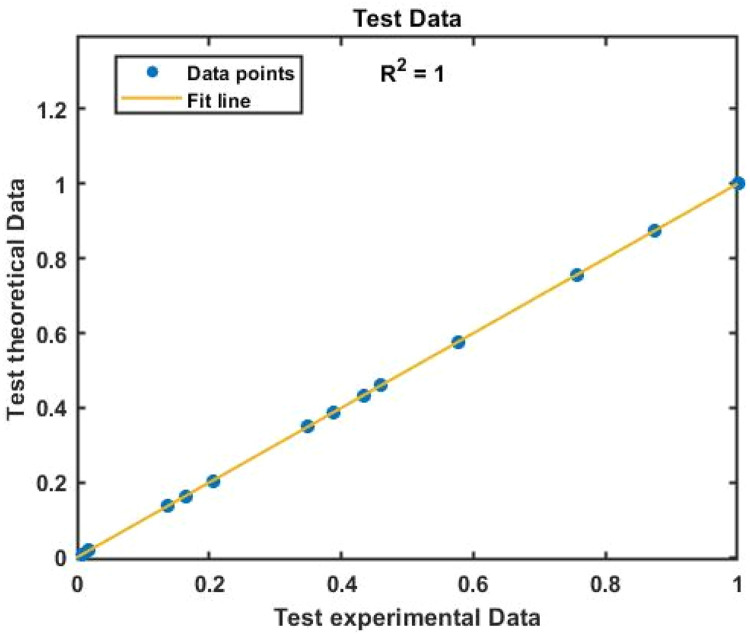
Experimental test data against theoretical test data.

**Fig. 8:**
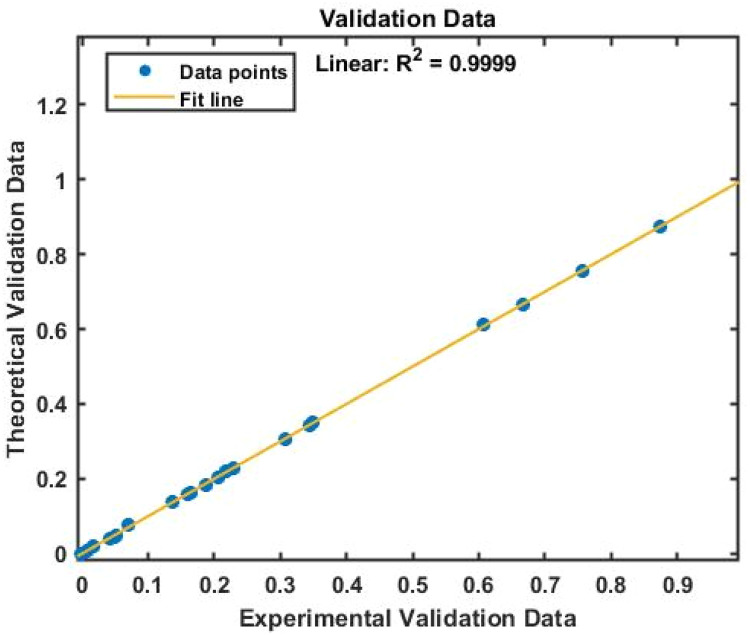
Experimental validation data against theoretical validation data.

**Fig. 9:**
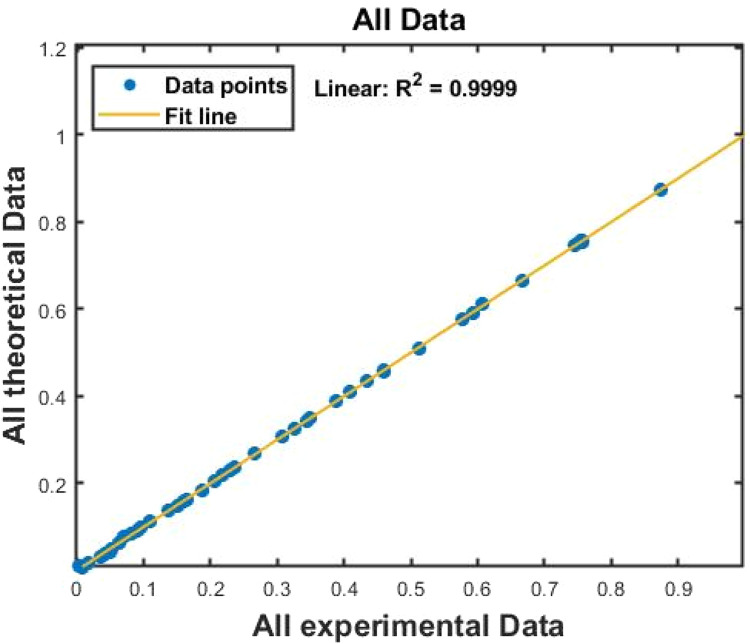
All experimental data against all theoretical data.

**Fig. 10:**
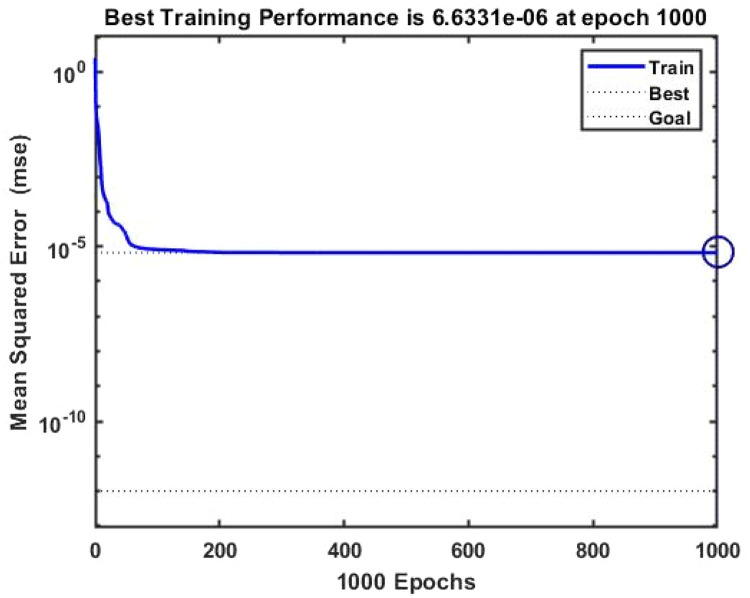
Training performance against mean square error.

**Fig. 11:**
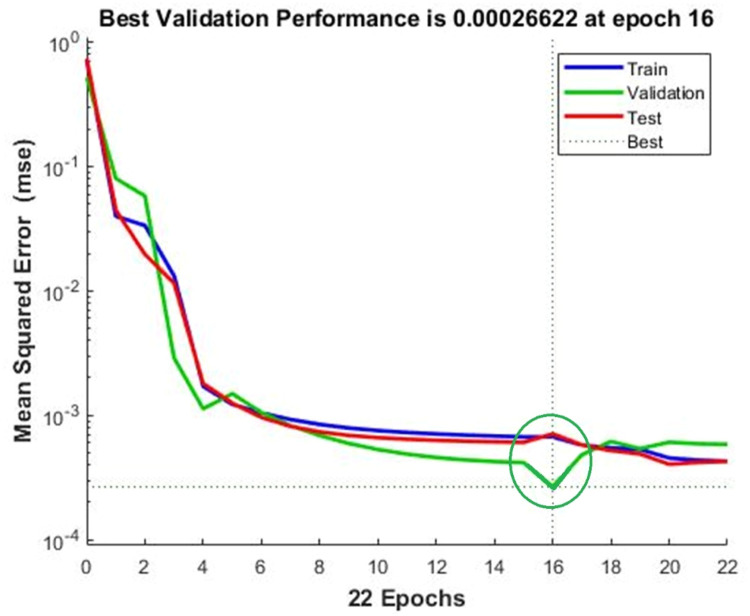
Performance diagram of viscosity output.

Fig. 6, Fig. 7 displays the viscosity of NiFe_2_O_4_−MWCNT Hybrid Nanofluid as a function of data number between the two experimental data and ANN results. The ANN accurately follows the experimental data. Thus, this ANN may be used to estimate the viscosity of the NiFe_2_O_4_−MWCNT nanofluid. Fig. 8 illustrates how the ANN results differ from the experimental data. The overall variation between experimental and theoretical data has been described in Fig. 9. Fig. 10 shows the variation in the viscosity of the NiFe_2_O_4_−MWCNT nanofluid vs the data number for ANN. The variance distribution in this figure is centered close to zero, indicating that the model is valid. Fig. 11 for NiFe_2_O_4_−MWCNT (30 - 70 %) / Oil 20W-50 hybrid nnanofluid. The vertical axis of this graph indicates the MSE, while the horizontal axis (Epoch) indicates the training iteration. This figure shows the MSE for the training, validation, and test points, respectively, for each distinct information set, comprising training, validation, and test. Fig. 12(a), Fig. 12(b) represent the variation of viscosity against data number using ANN and RSM respectively. The variation in the RSM model is greater than the ANN model. There are some variations for both methods, but Unlike the RSM model, the ANN model's error range is smaller. All these approaches have some variations, and the accuracy of the ANN model is greater than that of the RSM model.

**Fig. 12(a):**
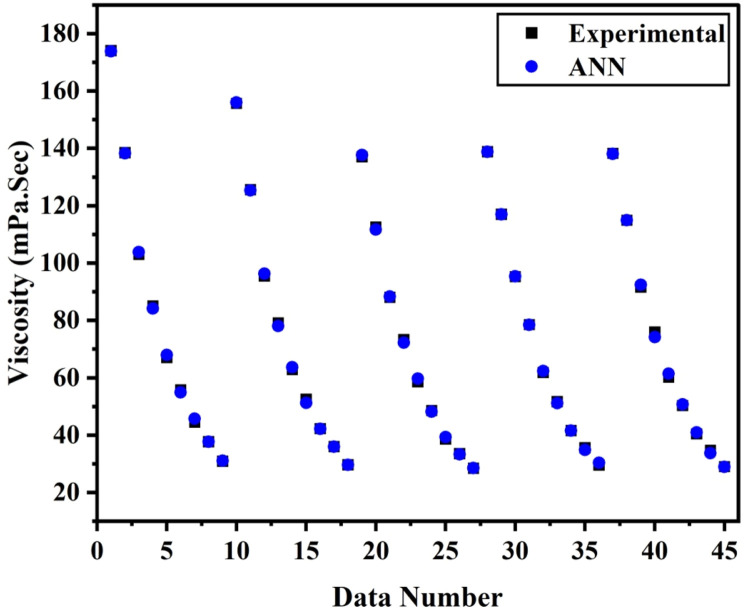
The viscosity of nanofluid against data number.

**Fig. 12(b):**
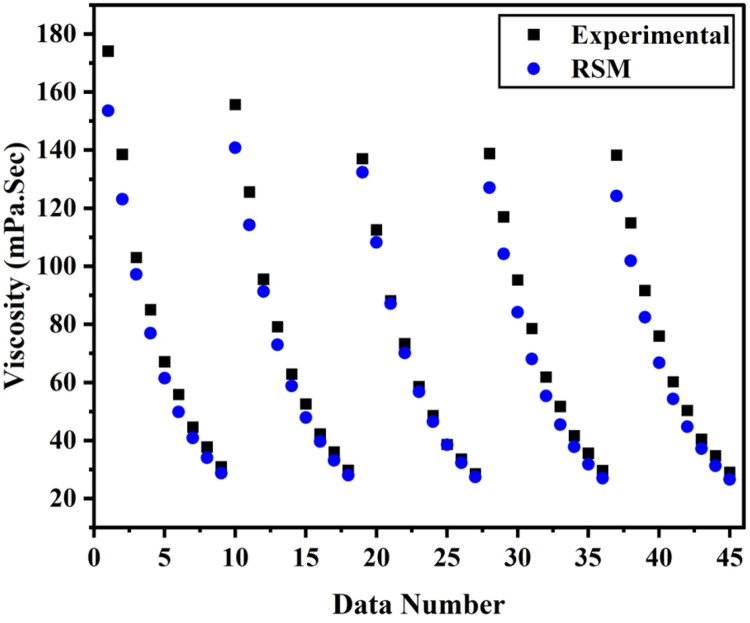
The viscosity of nanofluid vs. data number.

**Fig. 13:**
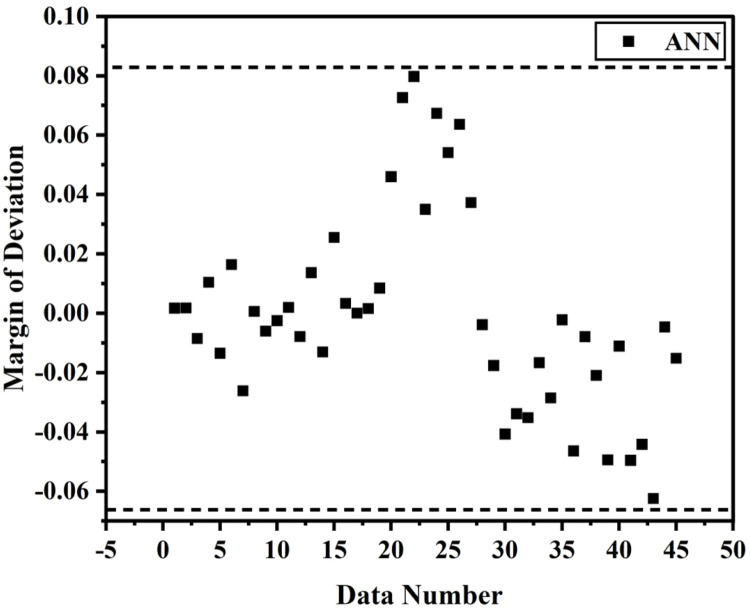
The accuracy of ANN results based on experimental measurements.

Equation 7 shows the margin of deviation (MOD), which compares the experimental data with the obtained ANN and RSM models. Fig. 13 demonstrates the agreement between results from experiments with ANN estimates. The highest reported margin of variation (MOD) percentages was −0.13 % and +0.04 %.

(5) $$MOD=1-\frac{\mu_{pred}}{\mu_{Exp}}$$

### Response surface methodology (RSM)

A nanofluid viscosity cannot be precisely predicted using straightforward theoretical formulae. Using the RSM model and assessing the experimental results led to the developing of a modified quadratic equation for calculating viscosity. Viscosity variations depending on volume concentration and temperature were predicted using experimental data. The experimental findings and the estimated relationship are in agreement, as shown by the theoretical correlation model's coefficient R^2^ of 0.9987.

(6) $$\frac{1}{\mu_{nf}}=\;\text{0.013393}+\text{0.001430}\varphi -\;\text{0.000612}T\;\text{+}\;\text{0.000030}\;\varphi T-\text{0.001093}\;\varphi ²+\text{0.000011}\;T²$$

The dynamic viscosity, volume concentration, and temperature are represented by μ_nf_, φ, and T, respectively. The proposed model with response surface method (RSM) methodology is correct, as shown by the analysis of variance (ANOVA) in tables [3] and [4].

High F-value (10,435.36) and low p value (smaller than 5 %) in Table 3 show that the parameters in Eq. (6) has high significance. Table 4,Table 5, Table 6

**Table 3:** ANOVA for nanofluids viscosity.

Source	Sum of Squares	df	Mean Square	F Value	P Value Prob> *F*	
**Model**	0.0012	5	0.0002	10,435.36	< 0.0001	significant
A-SVF	7.022 × 10^–06^	1	7.022 × 10^–06^	316.38	< 0.0001	
B-TEMP	0.0011	1	0.0011	49,343.92	< 0.0001	
AB	3.705 × 10^–07^	1	3.705 × 10^–07^	16.69	0.0047	
A²	2.062 × 10^–07^	1	2.062 × 10^–07^	9.29	0.0186	
B²	0.0000	1	0.0000	2237.60	< 0.0001	
**Residual**	1.554 × 10^–07^	7	2.220 × 10^–08^			
Lack of fit	1.554 × 10^–07^	3	5.179 × 10^–08^			
Pure Error	0.0000	4	0.0000			
**Cor Total**	0.0012	12

**Table 4:** Fit statistics.

Std. Dev	0.0001	R²	0.9999
Mean	0.0180	Adjusted R²	0.9998
C.V %	0.8262	Predicted R²	0.9987
PRESS	1.503 × 10^–06^	Adeq Precision	288.3503

**Table 5:** Model comparison statistics.

Source	Sequential p-value	Adjusted R²	Predicted R²	
Linear	< 0.0001	0.9420	0.9064	
2FI	0.8121	0.9359	0.8186	
**Quadratic**	**< 0.0001**	**0.9998**	**0.9987**	**Suggested**
Cubic	0.0090	1.0000	0.9976	**Aliased**

**Table 6:** Coefficient in terms of coded factor.

Source	Sum of Squares	df	Mean Square	F-value	p-value	
Mean vs Total	0.0042	1	0.0042			
Linear vs Mean	0.0011	2	0.0006	98.39	< 0.0001	
2FI vs Linear	3.705 × 10^–07^	1	3.705 × 10^–07^	0.0599	0.8121	
**Quadratic vs 2FI**	**0.0001**	**2**	**0.0000**	**1249.91**	**< 0.0001**	**Suggested**
Cubic vs Quadratic	1.318 × 10^–07^	2	6.590 × 10^–08^	13.98	0.0090	Aliased
Residual	2.356 × 10^–08^	5	4.713 × 10^–09^			
Total	0.0054	13	0.0004			

**Fig. 14:**
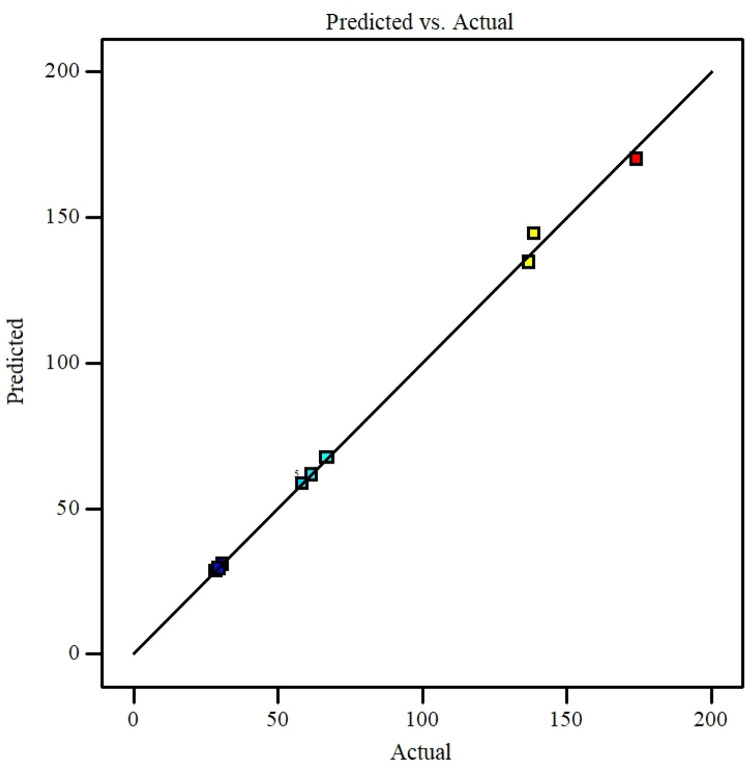
Results from experiments and predictions made using the correlation model equation for hybrid nanofluid.

**Fig. 15:**
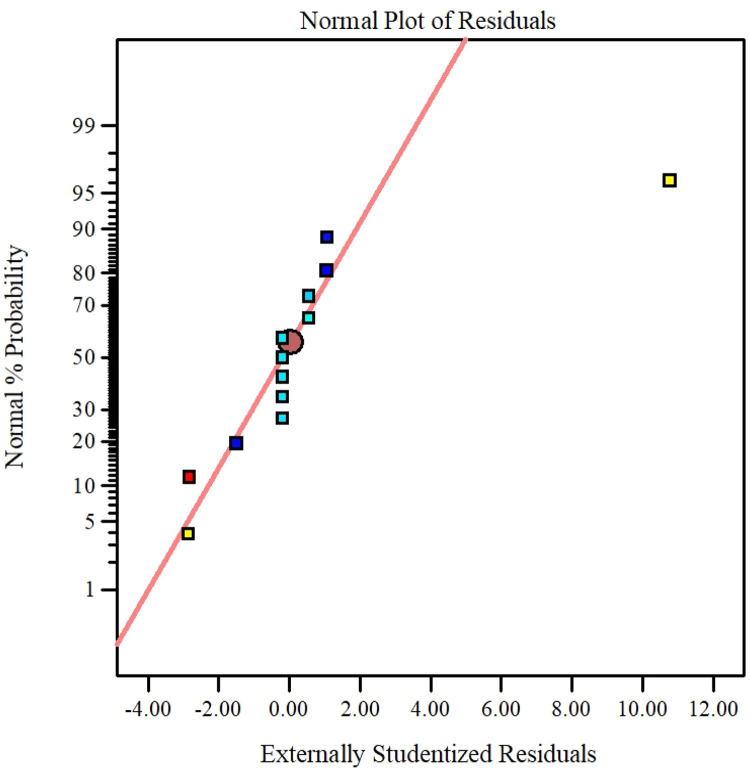
Residuals for the hybrid nanofluids viscosity vs. the normal probability curve.

**Fig. 16:**
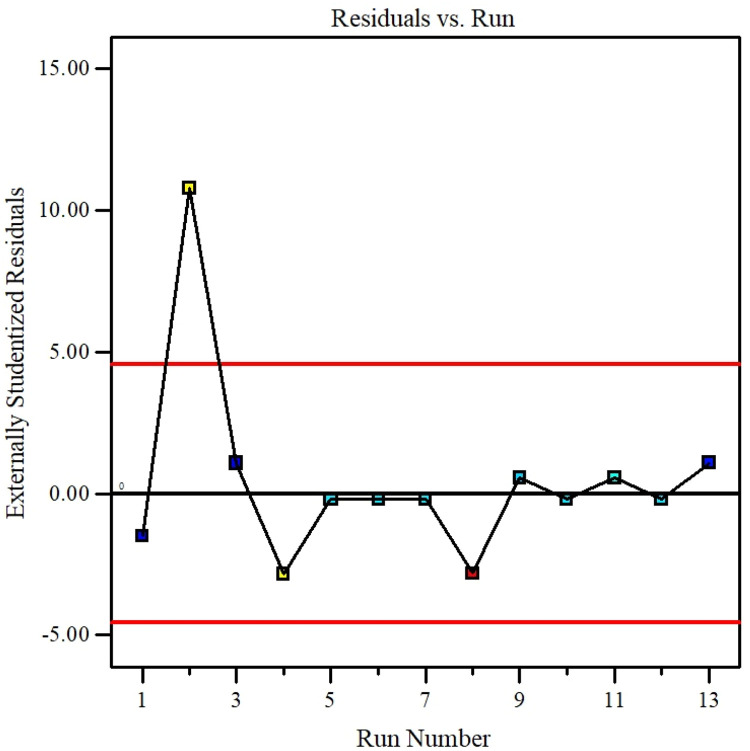
The CCD Viscosity prediction against externally studentized residuals.

**Fig. 17:**
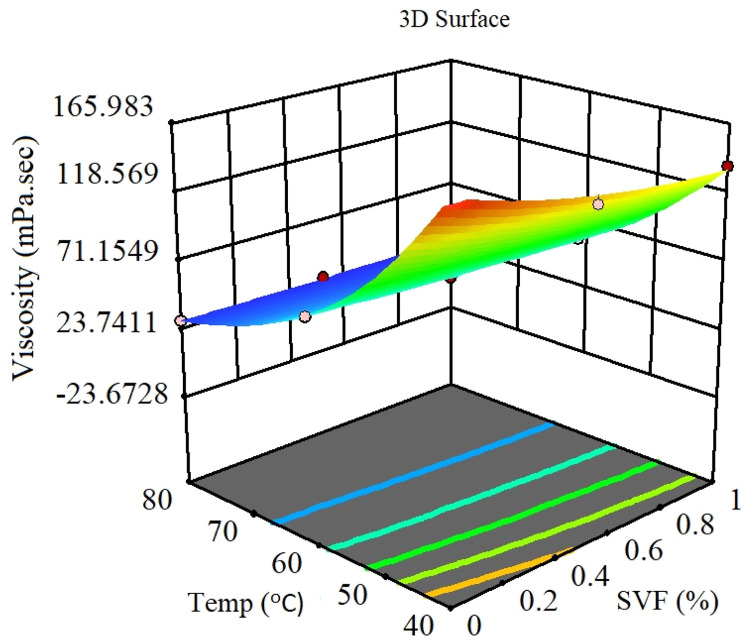
The hybrid nanofluid viscosity's Response Surface plot as influenced by the independent variable.

**Fig. 18:**
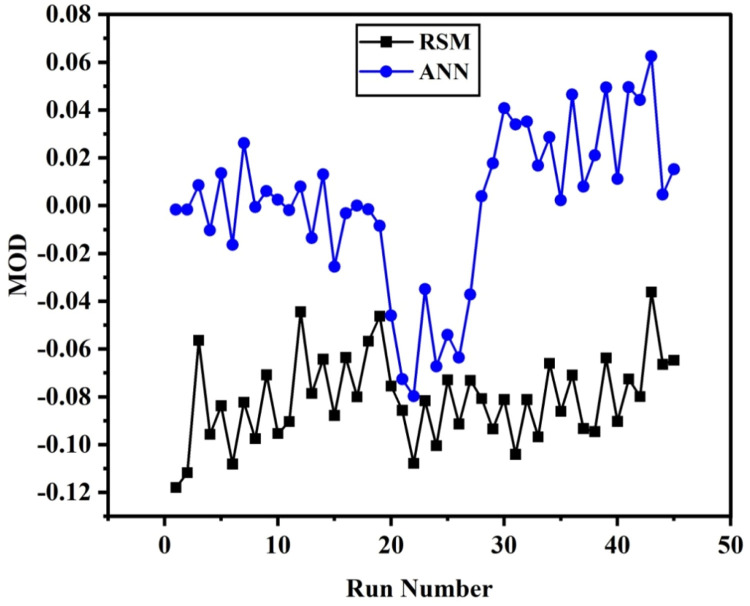
ANN and RSM variations on the viscosity of hybrid nanofluid against data number.

The parameter can be eliminated from the equation if the p-value exceeds five percent because it has little effect on the mathematical correlation. Experimental against predicted values described in Fig. 14 that shows the model accuracy. Residuals for the hybrid nanofluid viscosity against the normal probability curve described in Fig. 15. Fig. 16 describes the CCD Viscosity prediction against externally studentized residuals. The 3D surface display of the variables solid volume fraction, temperature and viscosity can be viewed in Fig. 17. The correlation technique, or RSM, may predict the experimental data in this Fig. 17; however, for specific data values, the difference between the actual and RSM values is not negligible. This approach is not as effective as the ANN method while being acceptable. Fig. 18 describes ANN and RSM variations on the viscosity of hybrid nanofluids against data number.

### Comparison with different studies

In the study, [21]modeling and optimization of thermal conductivity and viscosity of water-based spinel ferrite (MnFe2O4 / H2O) nanofluid under magnetic field using an ANN. In this work using the ANN, MSE values for the viscosity of the nanofluid was determined as 8.56 × 10–7 and 4.14 × 10–4 and correlation coefficient R2 was 0.996 and 0.994 for training data and test data respectively, which shows the significance of ANN model. In another study, [22] prediction of viscosity of cobalt ferrite/SAE50 engine oil based nanofluids using well trained Artificial Neutral Network (ANN) and Response Surface Methodology (RSM). In this work the coefficient of correlation R2 was determined as 0.9941 and 0.9663 using ANN and RSM respectively. In the current study presented the predictive modeling of the viscosity of NiFe2O4-MWCNT / SAE 20W-50 oil hybrid nanofluid was done. NiFe2O4 nano particles have diverse applications including catalysis, energy storage and biomedical fields, leveraging their unique magnetic properties. They have high specific capacity that’s why they are used in Lithium-ion batteries. Multi walled carbo nano tubes have high mechanical strength, thermal conductivity and electrical conductivity. The superiority of the study is that hybrid nano fluid was used for predictive modeling, which is very useful for the vehicles in warmer areas. The MSE values determined as 0.00001 and 0.0002 and R2 values 0.9999 and 0.9987 using ANN and RSM respectively.

### Comparison between ANN and RSM

Both the methods ANN and RSM we had used for the predictive modeling of viscosity of hybrid nanofluid (NiFe_2_O_4_−MWCNT/20w-50 engine oil). Both the method has significance at its own, but there is some accuracy difference between them. From R^2^ (0.9987 for RSM and 0.9999 for ANN) It can be concluded that ANN have more accuracy than RSM, also from Mean square error value the accuracy of ANN over RSM can be decided. MSE value for RSM 0.0002 has greater than that from ANN which is 0.00001. Fig. 12(a), Fig. 12(b) describes the comparison of experimental data with ANN and RSM predicted data respectively, which also shows the significance of ANN over RSM.

## Conclusion

The viscosity of hybrid NiFe_2_O_4_−MWCNT nano-particles dispersed in SAE 20W-50 engine oil has been studied in this study. The studies are conducted at temperatures ranging from 40 °C to 80 °C and volume concentrations between 0 % and 1 %. NiFe_2_O_4_ nano-particles with an average particle size of 20 nm are produced using the Sol-gel auto combustion method. We used scanning electron microscopy and X-ray diffraction techniques to ascertain the structure of the nano-particles. The XRD data indicates that the NiFe_2_O_4_ nano-particles have a cubical spinel structure. A falling ball viscometer is used to measure the viscosity of a nanofluid. Additionally, the experimental results show that temperature and volume concentrations directly affect viscosity. Additionally, some well-known theoretical models are compared to the experimental data. Temperature and volume concentration are two variables that must be empirically correlated to determine nanofluids’ viscosity. The viscosity of a nanofluid is a further significant attribute that significantly affects the transfer of heat and hence demands similar attention in future studies.

The most important results were as follows.

1. Temperature is a function of viscosity.
2. Due to hybrid nanofluid viscosity, the prepared nanofluid NiFe_2_O_4_−MWCNT (30–70 %) / Oil 20W–50 engine oil is best for vehicle engines in warm areas.
3. The coefficient of determination is found to be R^2^ values of 0.9987 and 0.9999 using RSM and ANN, respectively.
4. The MSE value using ANN is 0.00001 which is less than that of 0.0002 using RSM that shows the significance of ANN over RSM.

## Limitations

This study have the limitations

Ø ANN Need for large amount of labeled training data
Ø ANN method is Computationally intensive and resource consuming

## Ethics statements

The Authors declare that they have no conflict of interest and the research was conducted in an ethical and responsible manner. The use of artificial neural networks and response surface methodology in predictive modelling of the viscosity of nano fluid was performed with transparency and accountability. The data and methods used are original and have not been misrepresented. The authors take full responsibility for the accuracy and validity of results.

## Credit author statement

Muhammad Furqan has contributed in predictive modelling the experimental data using artificial neural network and response surface methodology and result and discussion section. Muhammad Khalid contributed in the formation of nanofluid and in the collection of experimental data of viscosity of nanofluid. Fahim Raees contributed in the numerical simulation, revising and supervising the project of the manuscript.

## Supplementary material *and/or* additional information [OPTIONAL]

Not Applicable.

## Declaration of competing interest

The authors declare that they have no known competing financial interests or personal relationships that could have appeared to influence the work reported in this paper.

## Data Availability

Data will be made available on request.
